# The First Case of Bovine Astrovirus-Associated Encephalitis in the Southern Hemisphere (Uruguay), Uncovers Evidence of Viral Introduction to the Americas From Europe

**DOI:** 10.3389/fmicb.2019.01240

**Published:** 2019-06-04

**Authors:** Federico Giannitti, Rubén Darío Caffarena, Patricia Pesavento, Francisco Alejandro Uzal, Leticia Maya, Martín Fraga, Rodney Colina, Matías Castells

**Affiliations:** ^1^Instituto Nacional de Investigación Agropecuaria (INIA), Plataforma de Investigación en Salud Animal, Estación Experimental INIA La Estanzuela, Colonia, Uruguay; ^2^Facultad de Veterinaria, Universidad de la República, Montevideo, Uruguay; ^3^Pathology, Microbiology and Immunology Department, School of Veterinary Medicine, University of California, Davis, Davis, CA, United States; ^4^Laboratorio de Virología Molecular, Centro Universitario Regional (CENUR) Litoral Norte, Universidad de la República, Salto, Uruguay

**Keywords:** bovine astrovirus, cattle, encephalitis, infectious diseases, *Mamastrovirus*, phylogeography, South America, Uruguay

## Abstract

Astrovirus species members of the *Mamastrovirus* genus (family *Astroviridae*) have been increasingly recognized as neuroinvasive pathogens in various mammals, including humans, mink, cattle, sheep, and pigs. While cases of astrovirus-associated encephalitis have been reported in North America, Europe, and Asia, their presence has never been documented in the Southern hemisphere. This paper describes a case of astrovirus-associated encephalitis in cattle in Uruguay that broadens the geographic distribution and genetic diversity of neuroinvasive astroviruses and provides phylogeographic evidence of viral introduction to the Americas from Europe. A 22-month-old Holstein steer from a farm in Colonia Department, Uruguay developed progressive neurological signs over a 3-days period before dying. Histopathological examination of the brain and proximal cervical spinal cord revealed disseminated, moderate to severe lymphocytic, histiocytic, and plasmacytic poliomeningoencephalomyelitis with neuronal necrosis. A *Mamastrovirus* strain in the CH13/NeuroS1 clade, that we called bovine astrovirus (BoAstV)-Neuro-Uy, was identified by reverse transcriptase PCR followed by nearly complete genome sequencing. Additionally, BoAstV was detected intralesionally in the brain by chromogenic RNA *in situ* hybridization within neuronal perikarya, axons and dendrites. Phylogenetic analysis of BoAstV-Neuro-Uy revealed a close relationship to neurotropic BoAstVs within the Virginia/Human-Mink-Ovine clade, which contains a growing cadre of neuroinvasive astroviruses. Analyzing the complete coding region of neuroinvasive BoAstVs sequences available in GenBank, we estimated an evolutionary rate of 4.27 × 10^-4^ (95% HPD 2.19–6.46 × 10^-4^) nucleotide substitutions/site/year. Phylogeographic analysis suggests that the common viral ancestor circulated in Europe between 1794–1940, and was introduced in Uruguay between 1849–1967, to later spread to North America and Japan.

## Introduction

The *Astroviridae* family contains non-enveloped, positive-sense, single-stranded RNA viruses within two genera, *Mamastrovirus* and *Avastrovirus*, which infect mammals and birds, respectively. Currently, the International Committee on Taxonomy of Viruses (International Committee on Taxonomy of Viruses [ICTV], 2018) recognizes 19 species, namely *Mamastrovirus-1* through *-19*, within the *Mamastrovirus* genus; however, there are numerous strains awaiting classification, some of which are tentatively considered new species (Donato and Vijaykrishna, 2017).

Since 2010, several astroviruses have increasingly been recognized as neuroinvasive in various mammalian species, including humans (Quan et al., 2010; Naccache et al., 2015), mink (Blomström et al., 2010), cattle (Li et al., 2013), sheep (Pfaff et al., 2017), and pigs (Boros et al., 2017). After initial recognition of bovine astrovirus-associated encephalitis in United States cattle (Li et al., 2013), a retrospective study in cases of sporadic bovine encephalitis of undetermined etiology from Switzerland revealed that this neuroinvasive astrovirus had gone undetected for decades (Selimovic-Hamza et al., 2016). Although the epidemiology and transmission routes of these astroviruses are unknown, cross-species transmission has been suggested based on the high level of identity (>98%), shared between bovine and ovine neuroinvasive astroviruses at the nucleotide and amino acid levels (Boujon et al., 2017).

Bovine astroviruses (BoAstVs), named BoAstV-NeuroS1 (Li et al., 2013) and BoAstV-CH13 (Bouzalas et al., 2014), were initially found in the brain of cattle with non-suppurative encephalitis in the United States and Switzerland, respectively. Despite the different nomenclature, both viruses represent the same genotype species (Bouzalas et al., 2016; Selimovic-Hamza et al., 2017a) that is still awaiting official classification by the ICTV. In 2015, a previously unknown BoAstV strain, named BoAstV-CH15, was identified in the brain of cows with encephalitis in Switzerland. Full genome phylogenetic comparison revealed a closer relationship of BoAstV-CH15 with an ovine astrovirus (OvAstV) than with BoAstV-CH13 (Seuberlich et al., 2016). Coinfection with BoAstV-CH13 and BoAstV-CH15 was also documented in one case (Seuberlich et al., 2016). The same year in Germany, Schlottau et al. (2016) reported a novel astrovirus, namely BoAstV-BH89/14, in a cow with encephalitis, that was most closely related to OvAstV and BoAstV-CH15. Subsequently, BoAstV-CH13/NeuroS1 was identified in 2017 in cases of bovine encephalitis in eastern and western Canada (Spinato et al., 2017; Selimovic-Hamza et al., 2017b). In 2018, a novel neuroinvasive BoAstV closely related with North American and European BoAstV-NeuroS1/BoAstV-CH13, was identified in a steer with non-suppurative encephalomyelitis in Japan, and the occurrence of intra-genotypic recombination between the North American and European strains was suggested (Hirashima et al., 2018).

While cases of astrovirus-associated encephalitis have been reported in North America, Europe, and Asia, their presence has never been documented in the Southern hemisphere. Here we describe a case of astrovirus-associated encephalitis in cattle in Uruguay, which broadens the geographic distribution and genetic diversity of neuroinvasive astroviruses and provide phylogeographic evidence that suggests that this virus was introduced into the Americas from Europe and later spread to Asia.

## Materials and Methods

### History and Signalment

In June 2018, a 22-month-old Holstein steer in a group of 37 steers in a ∼300-hectare farm in Colonia, Uruguay, developed progressive neurological sings including unusual behavior, aimless walking, circling, ataxia, repetitive and uncoordinated tongue movements, and recumbency. The herd grazed on an annual oat pasture and was supplemented with corn silage. A presumptive clinical diagnosis of cerebral listeriosis by the veterinary practitioner prompted treatment with penicillin and streptomycin, however the animal died spontaneously after a clinical course lasting 3 days.

### Pathologic Examination, *in situ* Hybridization (ISH) and Immunohistochemistry (IHC)

The head of the steer was removed from the carcass and submitted to INIA’s Veterinary Diagnostic Laboratory (Animal Health Platform) for diagnostic work-up. Half of the brain, a short segment of proximal cervical spinal cord (C1), trigeminal ganglion and root of the trigeminal nerve, salivary gland, retropharyngeal lymph node, oropharynx, esophagus, tongue and skeletal muscle, were immersion-fixed in 10% neutral buffered formalin for 48–72 h. Tissues were routinely processed for histology, embedded in paraffin, microtome-sectioned at 4–5 μm and stained with hematoxylin and eosin (H&E) and Gram stains.

Chromogenic ISH was performed manually on 5 μm sections of formalin-fixed, paraffin-embedded (FFPE) brainstem, cerebrum and cerebellum on Superfrost Plus slides (Thermo Fisher Scientific, Pittsburgh, PA, United States) using the RNAscope 2.5 Red assay kit (Cat #322360, Advanced Cell Diagnostics, Hayward, CA, United States) and the BoAstV probe Cat. #406921. The probe is composed of 20ZZ pairs targeting region 5232–6180 of the virus (GenBank KF233994.1). Each 5 μm section of tissue was pretreated with heat and protease prior to probe hybridization for 2 h at 40°C and processed as per the manufacturers’ recommendations. Negative controls used for validation of signal included an unrelated (GC-content matched) probe run on serial sections and probing tissue from uninfected animals. Slides were counterstained with hematoxylin and mounted with EcoMount (Biocare Medical, Concord, CA, United States).

Additionally, IHC was performed in FFPE sections of brainstem, cerebrum and cerebellum, as previously described, for the identification of West Nile virus (WNV, *Flavivirus*) (Palmieri et al., 2011), rabies virus (*Lyssavirus*) (Stein et al., 2010), and *Chlamydia* spp. (Giannitti et al., 2016) antigens.

### Molecular Virology

Nucleic acid extraction was accomplished from a pooled sample of frozen (-20°C) brain using MagMAX Nucleic Acid Isolation Kit^®^ (Thermo Fisher Scientific). For astrovirus detection, reverse transcription (RT) was performed with RevertAid Reverse Transcriptase^®^ (Thermo Fisher Scientific) and random hexamer primers (Qiagen). PCR was performed from cDNA using MangoMix^®^ (Bioline) and primers that amplify a 432-nucleotide fragment of the astrovirus polymerase gene (Tse et al., 2011). The PCR product was visualized in 2% agarose gel, purified using PureLink^®^ Quick Gel Extraction and PCR Purification Combo Kit (Invitrogen), and sequenced at Macrogen Inc. (Seoul, South Korea). For astrovirus whole genome amplification, Maxima H Minus Reverse Transcriptase (Thermo Fisher Scientific) and oligo(dT)18 for obtention of cDNA, and MangoMix^®^ (Bioline) or Ranger DNA Polymerase (Bioline) with primers described by Hirashima et al., 2018, were used. The PCR products were visualized in 1–2% agarose gel, purified and sequenced as mentioned above. Sequence assembly was conducted with SeqMan (Lasergene 8, DNASTAR). Twenty-six complete genome sequences of neuroinvasive astrovirus from cattle, sheep, pigs, humans and mink, and enteric bovine astrovirus available in GenBank were downloaded and aligned using Clustal W in MEGA 7 software (Kumar et al., 2016). W-IQ-TREE^1^ (Trifinopoulos et al., 2016) was used to determine the best-fit model of sequence evolution (SYM+I+G4) and to construct a maximum-likelihood phylogenetic tree with the nearly complete sequences of the BoAstV detected in this case, and those complete sequences downloaded from GenBank, using bootstrap as the statistical method to assess clades robustness. Similarity plot was performed with SimPlot software (Lole et al., 1999). *P*-distances at amino acid level of the ORF2 were estimated with MEGA 7 software (Kumar et al., 2016).

Additionally, a Bayesian phylogeographic analysis was performed with the BEAST v1.8.4 package (Drummond et al., 2012), using: the complete coding region of BoAstV CH13/NeuroS1 lineage, ORF1ab (non-structural genes), ORF2 (structural gene), ORF1a (protease) and a partial ORF1b (polymerase genomic region, for which Canadian strains were available), with all the sequences available in GenBank (last accession April 18, 2019), to determine the evolutionary rate, the ages/years of the common ancestors, and the most probable route of viral circulation by country (Switzerland, Uruguay, United States, Canada, and Japan). Lack of recombination in the dataset was determined using Recombination Detection Program 4. The substitution model that best fit each alignment was determined using MEGA 7 software through Bayesian information criterion (BIC) values, and the temporal structure of each dataset was evaluated using TempEst (Rambaut et al., 2016). The lognormal relaxed molecular clock with Bayesian Skyline analysis was selected by Bayes Factor among the different combinations of molecular clocks and coalescent tree priors used. The country of detection was used as trait. The Markov chain Monte Carlo length was 100 million generations, ensuring the convergence of the analysis, evaluated in Tracer v1.6.0, and the posterior probability was used to evaluate clades. The maximum clade credibility tree (MCCT) was obtained using TreeAnnotator software from BEAST and visualized in FigTree v1.4.3.

Lastly, DNA extracted from frozen brain was processed by PCR for the detection of bovine herpesviruses 1 and 5 (BHV-1 and -5), as previously described (Ashbaugh et al., 1997).

### Bacteriology

Fresh samples of cerebrum and brainstem were routinely processed for aerobic bacterial cultures in blood and MacConkey agars, and selective culture for *Listeria monocytogenes* (Al-Zoreky and Sandine, 1990).

## Results and Discussion

The clinical signs and epidemiological findings in the case described herein, albeit non-specific, were similar to those described in other cases of bovine astrovirus-associated encephalitis, which is usually described as sporadic (Selimovic-Hamza et al., 2016), with a variety of neurological deficits (Deiss et al., 2017), with a duration of clinical signs that typically ranges from 1 day to 3 weeks (Schlottau et al., 2016; Deiss et al., 2017; Spinato et al., 2017; Hirashima et al., 2018).

**FIGURE 1:**
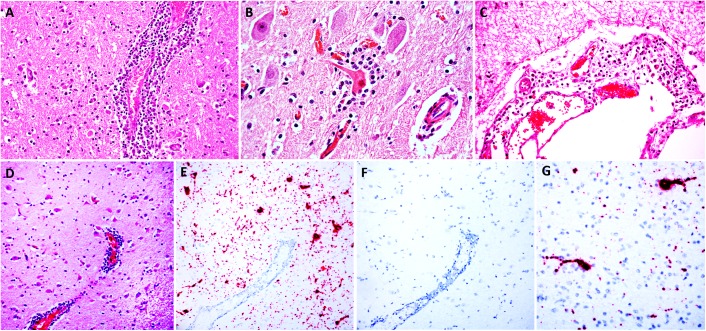
Histologic lesions in the brainstem **(A,B)** and cerebral cortex **(C,D)** and detection of BoAstV RNA in the cerebral cortex **(E,G)**. Images **(A–D)** are sections of brain stained with H&E; images E and G are sections of cerebral cortex demonstrating hybridization using chromogenic ISH using the BoAstV specific probe, counterstained with hematoxylin; image F is a serial section of cerebral cortex DapB probe (negative control), counterstained with hematoxylin. **(A)** A perivenular space is markedly expanded by inflammatory cells (mostly lymphocytes and histiocytes) that also infiltrate the adjacent neuropil. **(B)** The neuron in the center has hypereosinophilic perikaryon and karyorrhexis (necrosis) and the neuronal body is surrounded by increased numbers of glial (satellitosis) and inflammatory cells. **(C)** The leptomeninge is infiltrated by lymphocytes and histiocytes. **(D)** A region of cerebral cortex with multiple hypereosinophilic (necrotic) neurons and a large vessel with perivascular lymphocytic cuffs. **(E)** In a serial section of cerebral cortex, the abundant intracytoplasmic BoAstV RNA labeling is depicted by strong, granular red chromogen deposition within neuronal cytoplasm of the soma and neuronal extensions **(E,G)**, that is not present with hybridization using the negative control probe **(F)**.

Macroscopic examination of the brain, the C1 segment of the spinal cord, and other tissues of the head did not reveal significant gross anatomic lesions. Histologically, there was moderate to severe, lymphocytic, histiocytic and plasmacytic meningoencephalomyelitis affecting the telencephalon (including the cerebral hemisphere and hippocampus), brainstem, and the only examined segment of spinal cord. Lesions were predominantly distributed in the gray matter and limiting areas of white matter. In affected areas there was perivascular cuffing and lymphoplasmacytic and histiocytic inflammation and neuronal necrosis/neuronophagia with gliosis in the adjacent neuropil. There was satellitosis of affected, necrotic neurons (Figure 1A–D). The lesions were much less frequent and severe in the cerebellar parenchyma, although there was multifocal moderate cerebellar leptomeningitis. No intralesional bacteria were found with H&E and Gram stains. No significant histologic changes were found in the other examined tissues.

A neuroinvasive viral infection was suspected upon histologic examination of the central nervous system. Cattle with encephalitis are of concern because many ruminant neuropathogens are zoonotic (Cantile and Youssef, 2016); thus, a diagnosis of encephalitis should prompt extensive laboratory testing to screen for infectious agents when possible. In the case described herein, IHCs for WNV, rabies virus and *Chlamydia* spp., and PCR for BHV-1 and -5 were all negative, and no pathogenic bacteria were cultured from brain tissue. Because the steer was < 2 years old and no spongiform changes were observed in the brainstem, the animal was not tested for bovine spongiform encephalopathy (BSE), which is an exotic disease of adult cattle that has never been reported in Uruguay. Moreover, BSE is not inflammatory (Cantile and Youssef, 2016).

*In situ* hybridization was performed using a probe generated from BoAstV-NeuroS1, and there was probe hybridization abundant within, and limited to the cytoplasm of neurons in the cerebral hemisphere and hippocampus (Figure 1E–G). In these areas the probe hybridization colocalized with necrotic neurons and regions of gliosis, with no probe hybridization detectable in the glial cells, or inflammatory cells of the perivascular cuffs. No viral nucleic acid was detected by ISH in the cerebellum, which had only minimal inflammatory lesions in the parenchyma but moderate leptomeningitis, or the brainstem, including sections with severe inflammation. This means that, topographically, detection of viral distribution by ISH was more limited than encephalitis in the sections examined, which has occasionally been described in cases of BoAstV-CH13/NeuroS1-associated encephalitis in cattle (Selimovic-Hamza et al., 2017a,b). A reason for this occasional lack of viral RNA detection in lesioned areas of brain might be the detection limit of the ISH, or the clearance of the virus in inflamed areas of the brain by the time of death, as previously suggested (Selimovic-Hamza et al., 2017b). As expected, no probe hybridization was detected by ISH in the brain tissue used as negative control.

**FIGURE 2:**
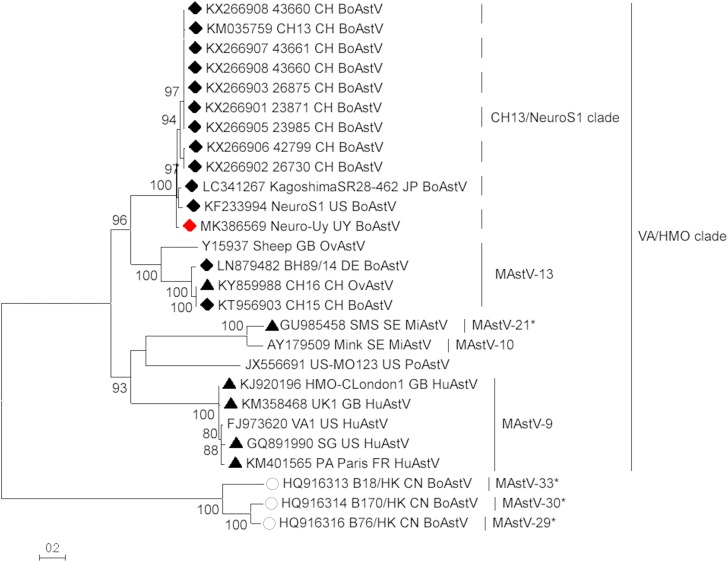
Phylogenetic analysis of full-length nucleotide sequences by maximum-likelihood method. BoAstV-Neuro-Uy is marked with a red rhombus. Other sequences of bovine neuroinvasive astroviruses are marked with black rhombi, sequences from non-bovine neuroinvasive astroviruses are marked with black triangles, and sequences from enteric bovine astroviruses are marked with white circles. *Mamastrovirus* species are shown; asterisks indicate species yet to be recognized by the ICTV. Strains within the CH13/NeuroS1 clade not yet assigned a species by the ICTV are identified with a dashed line. Bootstrap values are indicated in nodes. CH, Switzerland; JP, Japan; US, United States of America; UY, Uruguay; GB, United Kingdom of Great Britain; DE, Germany; SE, Sweden; FR, France; CN, China. BoAstV, bovine astrovirus; OvAstV, ovine astrovirus; PoAstV, porcine astrovirus; HuAstV, human astrovirus; MiAstV, mink astrovirus.

Astrovirus was detected in brain by RT-PCR. Nearly complete genome sequence analysis revealed a *Mamastrovirus* strain within the CH13/NeuroS1 clade, we named BoAstV-Neuro-Uy, the sequence was deposited in GenBank under accession number MK386569. The phylogenetic analysis revealed proximity with other neuroinvasive astroviruses within the Virginia/Human-Mink-Ovine (VA/HMO) clade (Figure 2), which contains most known neuroinvasive astroviruses (Hirashima et al., 2018; Reuter et al., 2018). The almost complete sequence of BoAstV-Neuro-Uy is 6427 bp in length and has a sequence identity of 94% with KagoshimaSR28-462 strain. BoAstV-Neuro-Uy has similar features as other strains of lineage CH13/NeuroS1: a 5′UTR region of 51 nt, ORF1a (protease) of 861 amino acids (aa), ORF1b of 523 aa (RNA-dependent RNA polymerase), and ORF2 of 758 aa (capsid protein). Unfortunately, the 3′UTR could not be sequenced, but a poly(A) tail is presumed to be present because oligo(dT)18 was used to obtain cDNA. In addition, the heptameric AAAAAAC sequence, a ribosomal frameshift signal, is present. *P*-distances at the amino acid level of the ORF2 confirmed the assignation of this strain to the CH13/NeuroS1 clade. *P*-distances < 0.35 between BoAstV-Neuro-Uy and other members of this clade (Table 1) would support a classification of these viral strains within one same species; *Mamastrovirus-13* has been recently proposed by other authors (Donato and Vijaykrishna, 2017; Hirashima et al., 2018), although definite species assignation by the ICTV is pending. The probe used for the ISH, generated from BoAstV-NeuroS1, had 92.7% sequence identity with BoAstV-Neuro-Uy.

**Table 1:** Estimates of evolutionary divergence at the amino acid level of the complete ORF2 region between sequences of bovine and ovine *Mamastrovirus-13* and strains within the CH13/NeuroS1 clade not yet assigned to a species by the International Committee on Taxonomy of Viruses (ICTV).

Sequences and GenBank accession numbers	1	2	3	4	5	6	7	8	9	10	11	12	13	14	15	16
1- KY859988_OvAstV_CH16	–	–	–	–	–	–	–	–	–	–	–	–	–	–	–	–
2- Y15937_OvAstV_OvAstV-1	0.263	–	–	–	–	–	–	–	–	–	–	–	–	–	–	–
3- LC341267_BoAstV_KagoshimaSR28-462	0.343	0.317	–	–	–	–	–	–	–	–	–	–	–	–	–	–
4- KM035759_BoAstV_CH13	0.341	0.321	0.012	–	–	–	–	–	–	–	–	–	–	–	–	–
5- KF233994__BoAstV_NeuroS1	0.348	0.327	0.016	0.015	–	–	–	–	–	–	–	–	–	–	–	–
6- KX266902_BoAstV_26730	0.341	0.321	0.012	0.005	0.015	–	–	–	–	–	–	–	–	–	–	–
7- KX266903_BoAstV_26875	0.344	0.320	0.012	0.005	0.015	0.005	–	–	–	–	–	–	–	–	–	–
8- KX266907_BoAstV_43661	0.341	0.320	0.009	0.003	0.012	0.003	0.003	–	–	–	–	–	–	–	–	–
9- KX266901_BoAstV_23871	0.343	0.321	0.012	0.005	0.012	0.005	0.005	0.003	–	–	–	–	–	–	–	–
10- KX266906_BoAstV_42799	0.341	0.320	0.011	0.004	0.013	0.004	0.004	0.001	0.004	–	–	–	–	–	-	–
11- KX266908_BoAstV_43660	0.341	0.320	0.011	0.004	0.013	0.004	0.004	0.001	0.004	0.003	–	–	–	–	–	–
12- KX266905_BoAstV_23985	0.341	0.318	0.012	0.005	0.015	0.005	0.005	0.003	0.005	0.004	0.004	–	–	–	–	–
13- KX266904_BoAstV_36716	0.341	0.320	0.008	0.004	0.013	0.004	0.004	0.001	0.004	0.003	0.003	0.004	–	–	–	–
14- MK386569_BoAstV_Neuro-Uy	0.342	0.323	0.016	0.013	0.019	0.013	0.013	0.011	0.013	0.012	0.012	0.013	0.012	–	–	–
15- KT956903_BoAstV_CH15	0.007	0.266	0.347	0.345	0.352	0.345	0.347	0.345	0.347	0.345	0.345	0.345	0.345	0.346	–	–
16- LN879482_BoAstV_BH89/14	0.007	0.260	0.344	0.343	0.349	0.343	0.344	0.343	0.344	0.343	0.343	0.343	0.343	0.344	0.011	–


*Based on current ICTV criteria, strains with *p*-distances < 0.35 should be considered within the same genotype species. The number of amino acid differences per site between sequences are shown. The analysis involved 16 amino acid sequences. All ambiguous positions were removed for each sequence pair. There was a total of 773 positions in the final dataset. OvAstV, ovine astrovirus; BoAstV, bovine astrovirus.*

Studies based on neuropathological examinations and astrovirus nucleic acid and protein detection have concluded that there is a probable causal relationship between astrovirus infection and neurological disease and lesions in cattle (Selimovic-Hamza et al., 2017a; Reuter et al., 2018). To the best of our knowledge astrovirus-associated encephalitis has not been reproduced experimentally yet. This would require isolation of neuroinvasive astroviruses from clinical cases, which was not attempted in our case.

The source of BoAstV-Neuro-Uy in this case could not be determined. However, reservoir cattle and wildlife should be considered, as the cattle were raised under extensive outdoor conditions. The affected animal had been purchased and moved to the farm in February 2018 along with other 9 steers. Unfortunately, the owner refused further sampling and testing of other animals in the property, and a more detailed epidemiological investigation. None of the other animals in the group had developed neurological disease as of August 2018, the last time the veterinary practitioner was contacted. A seasonality from the beginning of winter until the end of the spring has been suggested for cases of astrovirus-associated encephalitis in Switzerland (Selimovic-Hamza et al., 2016). Interestingly, the case described herein occurred in June, corresponding to the autumn-winter transitional period in the Southern hemisphere.

While neurotropic astroviruses have been identified in North America (Li et al., 2013; Spinato et al., 2017), Europe (Bouzalas et al., 2014) and Asia (Hirashima et al., 2018), their presence has never been reported in the Southern hemisphere, so this communication broadens the geographic distribution of astrovirus-associated encephalitis. To assess whether the viral strain detected in Uruguay might have originated in Europe, North America, or Asia, we estimated the evolutionary rate and performed a phylogeographic analysis using neuroinvasive BoAstV sequences available in GenBank. The evolutionary rate estimated using the complete coding region was 4.27 × 10^-4^ (95% highest probability density -HPD-, 2.19–6.46 × 10^-4^) nucleotide substitutions/site/year, which is expected for an RNA virus (Jenkins et al., 2002), but lower than that estimated for enteric human astroviruses (Babkin et al., 2012, 2014). The ORF1ab region showed a similar evolutionary rate (4.20 × 10^-4^, 95% HPD 1.66–6.46 × 10^-4^ substitutions/site/year) as the complete coding region, while the ORF1a (2.92 × 10^-4^, 95% HPD 1.19 × 10^-6^–6.46 × 10^-4^ substitutions/site/year) and ORF2 (2.86 × 10^-4^, 95% HPD 4.13 × 10^-6^–5.79 × 10^-4^ substitutions/site/year) showed a slightly faster evolutionary rate, and the partial polymerase genomic region (ORF1b) showed a slightly slower evolutionary rate (5.39 × 10^-4^, 95% HPD 6.41 × 10^-7^–1.10 × 10^-3^ substitutions/site/year).

**FIGURE 3:**
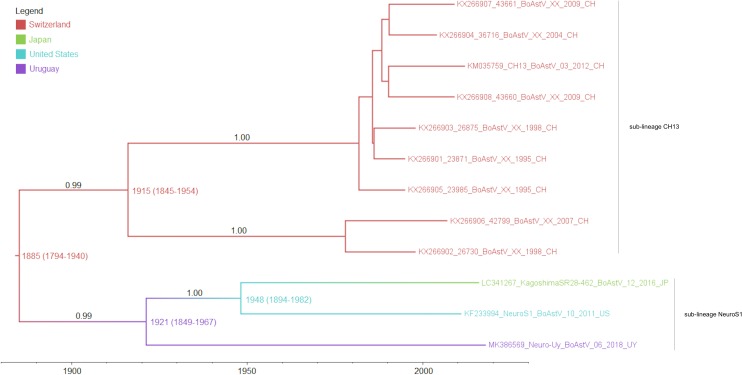
Maximum clade credibility tree obtained by analysis of the full-length coding region. The color of the branches represents the most likely country where the ancestors circulated, posterior probability values are shown in the branches and the numbers in each node represent the years of origin for each clade with the 95% HPD interval. Sub-lineages are indicated with labels.

As determined by the phylogeographic analysis with the complete coding region, and shown in the MCTT (Figure 3), there are two sub-lineages (CH13 and NeuroS1) based on reference strains, that have a common ancestor. The most recent common ancestor of these sub-lineages (lineage CH13/NeuroS1) arose in Europe approximately in 1885 (95% HPD, 1794–1940). At the beginning of the 1900’s, the two sub-lineages diverged, the CH13 sub-lineage stayed circulating in Europe, while the NeuroS1 sub-lineage spread to America and Asia. The most likely scenario is that the NeuroS1 sub-lineage was introduced in Uruguay from Europe around the year 1921 (95% HPD, 1849–1967), presumably through livestock trade, then spread to North America, and later to Japan (Figure 3). Due to the limitation in the number of sequences available in GenBank, which could have biased the analysis, the results obtained using the complete coding region were compared with those obtained with other genomic regions (ORF1ab, ORF2, ORF1a, and ORF1b) available for a larger number of strains (i.e., Canadian strains). In all the analyses the most likely scenario is that the introduction of the virus to Uruguay occurred from Europe (Supplementary Figures S1A–D). In addition, the estimated date for this introduction, obtained with the ORF1ab and partial polymerase genomic region (ORF1b) (Supplementary Figures S1A,D), was similar to that obtained with the complete coding region, whereas the estimated date of introduction obtained with ORF2 and ORF1a was earlier but with wider 95% HPD interval (Supplementary Figures S1B,C). An introduction of the sub-lineage NeuroS1 directly to Canada from Europe, with subsequent spread to United States and Japan, is also plausible, s shown in Supplementary Figure S1D.

Further investigations are needed to assess the geographic distribution, pathogenic mechanisms (particularly mechanisms of transmission and entry), molecular epidemiology, and potential interspecies transmission of neuroinvasive astroviruses.

## Data Availability

The datasets generated for this study can be found in GenBank, MK386569.

## Author Contributions

FG, RDC, and MC contributed with the conception of the study. FG and RDC performed the pathological examination and sampling. PP performed the *in situ* hybridization. FU performed the immunohistochemistry. LM, RC, and MC performed molecular virology testing. MC performed the sequence and phylogeographic analyses and associated figures. FG and PP obtained the histologic images. MF performed the bacterial cultures. FG and MC wrote the first draft of the manuscript. RDC, PP, FU, LM, MF, and RC wrote sections of the manuscript. All authors contributed to manuscript revision, read and approved the submitted version.

## Conflict of Interest Statement

The authors declare that the research was conducted in the absence of any commercial or financial relationships that could be construed as a potential conflict of interest. The reviewer TS declared a past co-authorship with one of the authors PP to the handling editor.

## Footnotes

1 http://iqtree.cibiv.univie.ac.at

## Supplementary Material

FIGURE S1 Maximum clade credibility trees (MCCTs) obtained by analysis of the full-length ORF1ab **(A)**, full-length ORF2 **(B)**, full-length ORF1a **(C)**, and partial ORF1b **(D)**. The color of the branches represents the most likely country where the ancestors circulated, posterior probability values are shown in the branches, and the numbers in each node represent the years of origin for each clade with the 95% HPD interval. Sub-lineages are indicated with labels.
