# Supplementary figures and images for: The First Case of Bovine Astrovirus-Associated Encephalitis in the Southern Hemisphere (Uruguay), Uncovers Evidence of Viral Introduction to the Americas From Europe
Source: Front Microbiol. 2019 Jun 4;10:1240. doi: 10.3389/fmicb.2019.01240 (PMC6559012; doi:10.3389/fmicb.2019.01240)

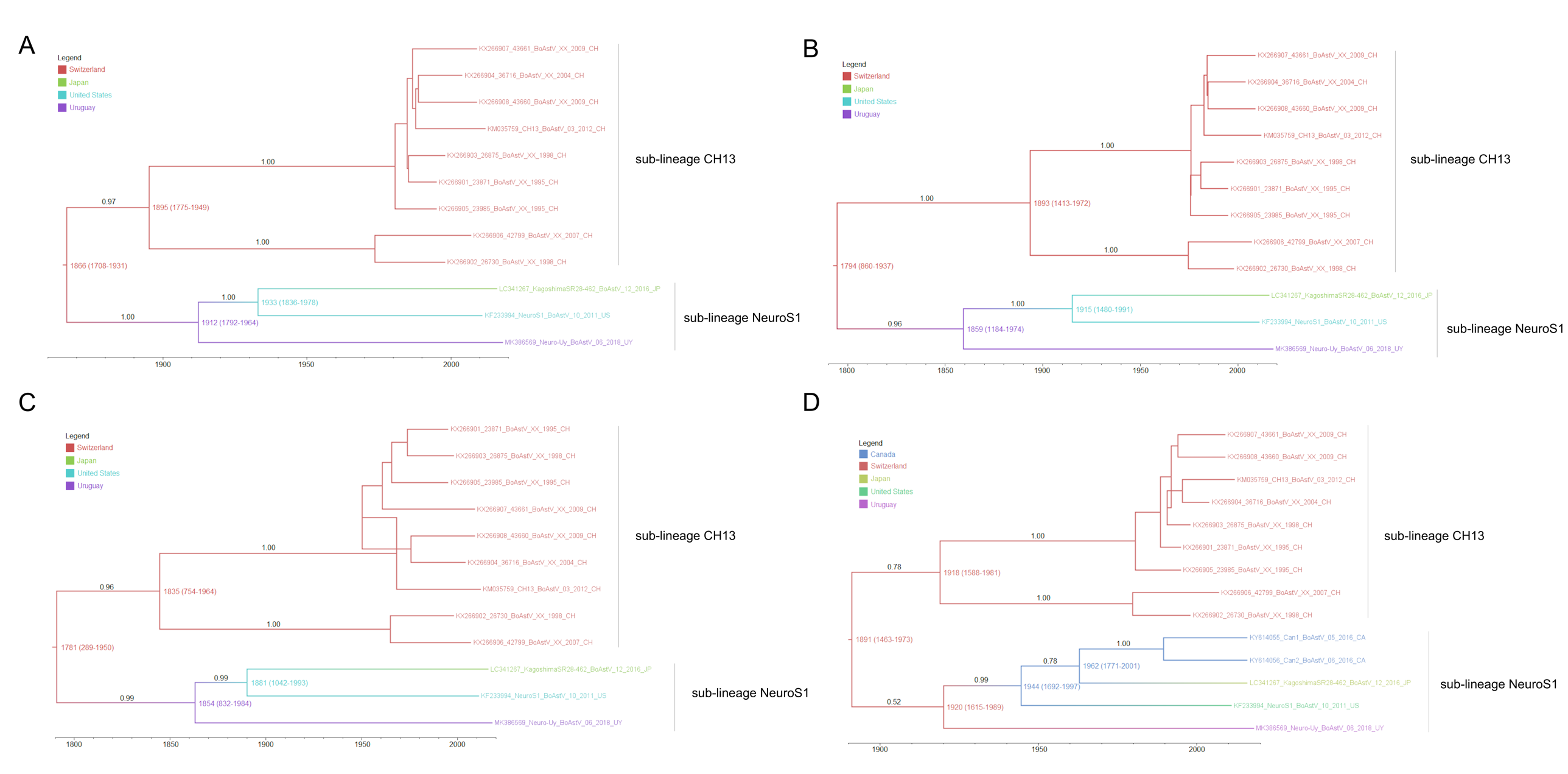

Supplement: FIGURE S1 — Maximum clade credibility trees (MCCTs) obtained by analysis of the full-length ORF1ab (A), full-length ORF2 (B), full-length ORF1a (C), and partial ORF1b (D). The color of the branches represents the most likely country where the ancestors circulated, posterior probability values are shown in the branches, and the numbers in each node represent the years of origin for each clade with the 95% HPD interval. Sub-lineages are indicated with labels. [file Image_1.TIFF]
